# The European Health Data Space: an opportunity to strengthen citizen rights and engage citizens in health data governance

**DOI:** 10.3389/fmed.2025.1699941

**Published:** 2026-01-20

**Authors:** Patricia Cervera de la Cruz, Teodora Lalova-Spinks, Mahsa Shabani

**Affiliations:** 1 Faculty of Law and Criminology, Ghent University, Ghent, Belgium; 2 Faculty of Medicine and Health Sciences, Ghent University, Ghent, Belgium; 3 Faculty of Law, University of Amsterdam, Amsterdam, Netherlands

**Keywords:** citizen engagement, citizen rights, European Health Data Space (EDHS), health data, implementation, secondary use

## Abstract

**Introduction:**

The European Health Data Space (EHDS), the European Union’s new regulatory framework for health data use and reuse, will have important implications for citizens across the Union. While the regulation aims to empower citizens in the primary use of their health data-such as by giving them access to their electronic health records-their role in the secondary use of health data remains less clearly defined.

**Methods:**

To explore this, we interviewed health data experts across 23 European countries to understand their perspectives on citizen involvement in data reuse.

**Results:**

Our findings reveal that while the provision for an opt-out mechanism provides individuals with control, its practical implementation requires careful design to ensure accessibility and operability. Experts also emphasized the importance of broader citizen engagement, both to raise awareness about the EHDS and to incorporate citizens’ perspectives into governance structures. Furthermore, clearly demonstrating the value of the EHDS was perceived to be crucial for fostering public trust and acceptance.

**Discussion:**

The study provides valuable insights for Member States in developing strategies to engage citizens in the secondary use of health data under the EHDS framework. It highlights the importance of an easily accessible opt-out mechanism, reinforced by clear and effective communication to prevent misinformation, emphasize the tangible benefits of data sharing, and account for the influence of institutional trust and country-specific contexts in shaping public attitudes toward the EHDS.

## 1 Introduction

The European Health Data Space (EHDS) (1) is a new EU regulation which sets rules for the use and reuse of health data across all Member States (MS). A key aspect of this regulation is the secondary use of health data—its reuse for purposes beyond individual care—which is crucial for advancing research and innovation in healthcare (2, 3). Artificial intelligence (AI) tools designed to be used in healthcare settings, for instance, require vast amounts of diverse health data to be properly validated and ensure representativeness. Furthermore, having access to health datasets is also critical for evaluating the impact of health policies and improving the efficiency of clinical trials (4), among other things. By mandating that health data holders share data within secure processing environments (EHDS, Article 73) and through Health Data Access Bodies (HDAB), (EHDS, Article 55) the EHDS aims to facilitate these exchanges while ensuring security and oversight.

Much of the discussion on the regulation, especially regarding the primary use purposes, has claimed that the EHDS “will empower individuals to take control of their health data” (5). This claim seems to be supported by certain provisions for the primary use of health data in the regulation such as Article 3, which grants citizens access to their electronic health records or Article 7, which allows them to request the transfer of their health data across borders. In turn, in the secondary use of health data—which also involves the use of citizens’ data—it seems equally important to allow individuals to be engaged in decisions about how their data is used for secondary purposes.

A citizen-centered approach to health data governance has been encouraged to ensure that citizens feel valued and included in conversations and decision-making about the secondary use of their health data. (6) However, there remains ambiguity regarding the extent and manner of citizen involvement in control and oversight of health data reuse. While some communities have grown distrustful of researchers due to past data use without their knowledge and involvement (7–9) and expect to remain in control over their data (10, 11), other empirical evidence suggests that many citizens are not always necessarily interested in direct involvement or control—provided proper safety measures are in place (12, 13).

In the EHDS, with regards to the secondary use, citizens, at an individual-level, are primarily granted control over their health data through the right to opt-out. (EHDS, Article 71) This right gives individuals the choice to not have their health data processed for secondary use purposes, with the exception that this opt-out may be overridden on a MS level for purposes with strong links to the public interest (EHDS, Recital 54). The opt-out was initially not included in the EHDS proposal because of concerns about the costs of relying on consent, as described in the impact assessment report accompanying the proposal (14). This approach sparked intense criticism from stakeholders, who argued that it failed to respect individuals’ rights (15–19). Critics argued that the EHDS proposal’s interpretation of consent as being burdensome and costly overlooked the value of a consent model and did not adequately consider possible alternatives such as broad consent or dynamic consent (20), which have been widely discussed in the literature (20–22) and supported by empirical evidence (23–25). Moreover, some critics also argued that an opt-in approach might be necessary (26), especially for certain types of health data, such as genetic data or sexual and reproductive health data, which they interpret to be more sensitive and/or at greater risk of reidentification (27).

Following this intense debate (28), the final regulation introduced the right to opt-out under Article 71 and gives MS the option to introduce rules and specific additional safeguards (which may include an opt-in) to certain types of health data, such as genetic data (EHDS, Recital 18). However, beyond the opt-out, which offers individual control to citizens, many questions remain surrounding citizen engagement under the EHDS, such as how information should be communicated to citizens, how to operationalize the opt-out or how to engage citizens at a collective level through governance structures, such as HDABs.

We argue that with the implementation of the EHDS, there is a renewed opportunity to discuss the effective engagement of citizens in the secondary use of their health data. This study examines how health data experts perceive the role of citizens in light of the upcoming regulation—how they should be engaged and what mechanisms could empower them to exercise meaningful control over their data.

## 2 Materials and methods

### 2.1 Study design

#### 2.1.1 Data collection

The study team developed a semi-structured interview guide (Supplementary Material 1). The interview guide was pilot-tested with one of the co-authors to assess content and understandability. Feedback from this pilot led to a shortening of the guide, improvements in the flow between sections, including the reordering of several questions and the reformulation of a few questions. The interviews were all conducted by PC, a female PhD candidate with training in qualitative research methods, with the presence of MS for some interviews. All interviews were performed between March 2024 and August 2024. All interviews were conducted in English. Interviews were audio and video recorded via Microsoft Teams and transcribed ad verbatim, with potentially identifying information eliminated to ensure confidentiality. The planned length of interview was one hour, with the average interview time being 50 min. Written informed consent was sought from all participants.

The study was approved by the Ethical Committee at the Faculty of Law and Criminology at Ghent University.

### 2.2 Research team

The research team consisted of three persons: PC, MS, and TLS. PC, MS and TLS designed the interview guide, which was informed by the authors’ prior studies (29, 30) and on the regulatory discussions taking place at the time PC and MS conducted the interviews. Transcripts were coded by at least two members of the research team, and discussed in periodic meetings with all three researchers.

### 2.3 Participants and recruitment

**FIGURE 1:**
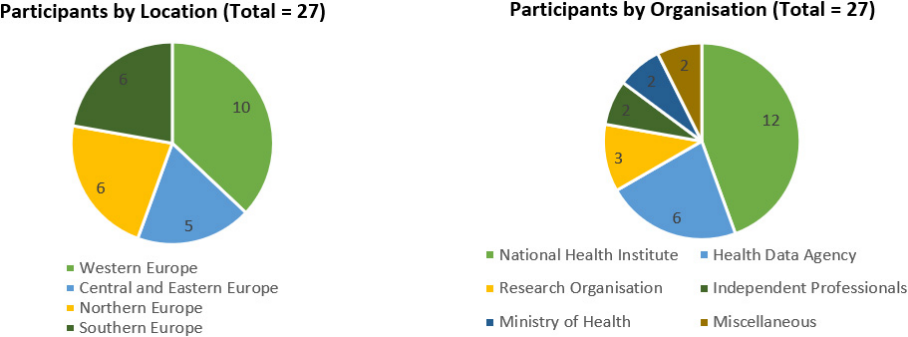
Overview of the interviewees by location and organization.

**FIGURE 2:**
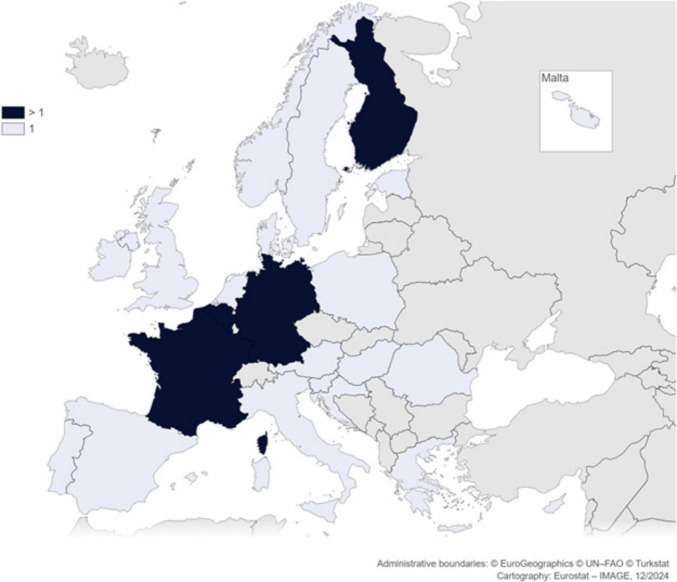
Map of participants’ geographical distribution.

We conducted interviews with 27 experts involved in the secondary use of health data in 23 different European countries, covering all four regions of Europe as defined by EuroVoc (31), ensuring comprehensive continental coverage, as shown in Figures 1, 2. Most of the interviews were one-to-one. For one interview, three participants from the same institution requested to be interviewed together, which was accommodated. We relied on purposive and snowball sampling.

### 2.4 Analysis

To analyze the data, we relied primarily on thematic analysis. We performed an inductive content analysis, where the content categories are derived from the data, rather than pre-determined (32, 33). Transcripts were coded into narrow content categories in NVivo 14 software by Lumivero. The initial coding categories informed the analysis, but for clarity and conciseness the subsections in the Results reflect higher-level themes that combine several of these categories rather than directly mirror the original coding framework. The first part of the results, which reveal health data experts’ expectations regarding the implementation of the EHDS, have been reported in a separate paper (34).

## 3 Results

The results of our study describe the views of health data experts regarding citizen engagement and empowerment under the EHDS. These issues were raised in the discussions about the opt-out mechanism, the obligations for citizen engagement under the regulation and the importance of demonstrating the value created by the EHDS for citizens.

### 3.1 Opt-out: empowering citizens, navigating implementation challenges, and ensuring effective communication

Many participants view the opt-out as a significant step in respecting citizens’ rights and empowering them. By providing an option to exercise control over their data, participants believe that the opt-out can help to foster a sense of agency among individuals.

“(…) [opt-out] is a safeguard for people to understand that they have rights and they can exercise it whenever they want” (P25).

However, participants also recognize that the opt-out represents a fundamental change from traditional practices, where consent has typically been sought from citizens. Conversely, in countries where certain types of health data have historically been collected without individuals’ explicit consent, participants noted that the opt-out introduces new rights for citizens.

Additionally, many welcomed the opt-out for its potential to improve the representativeness of health data, reducing selection biases, presuming a majority will not opt-out. However, concerns remained about a large number of opt-outs, for others.

“You want people not even to think about opting out because opting out is of course always a threat for research. And if the research is not based on a sample that is representative or even exhaustive for the whole population, you cannot do anything with your research results. So the opt-out at a massive scale is a big a threat for research” (P21).

Participants emphasized that the success of the opt-out system hinges on how it will be implemented. They stressed the importance of making it truly understandable and accessible, often drawing comparisons to the opt-out system for organ donation, which some found inadequate due to the difficulty in exercising the option.

“If you really believe in opt-out, you need to inform people and give them that right. There is no right to opt-out if you create a little software somewhere but you never tell people about it” (P8).

Participants also recognized that cultural sensitivities toward data protection and consent could significantly influence the opt-out’s acceptance. In some countries, historical distrust in institutions could lead to higher opt-out rates. Striking the right balance in communicating the opt-out was seen as a challenge. Some worried that excessive publicity could result in a surge of opt-outs. As one participant put it, the opt-out should be:

“(…) accessible, not too complicated, not too much in your face either” (P20).

Views were mixed on the level of granularity the opt-out should offer. Some questioned the feasibility of allowing citizens to opt-out at the level of specific research questions or themes, while others emphasized that a granular approach would be necessary to comply with the EHDS provision allowing individuals to request not to be informed of clinically significant findings. Participants also highlighted the technical challenges of implementing a national opt-out system, emphasizing the need for a centralized mechanism that ensures individuals only have to opt-out once, with this choice being reliably recorded and recognized across all national databases.

Reflecting on their own experiences, participants suggested ways to improve implementation. Some advocated for every citizen to receive a physical letter, while others favored digital solutions, such as integrating the opt-out into existing health information platforms. One participant noted that in their country, citizens can already opt-out of primary use via a digital platform, with an available alternative physical office for those unable or unwilling to opt-out online.

Many participants expressed concerns that misinformation campaigns could heavily influence citizens’ decisions to opt-out, pointing to cases where similar misinformation had already triggered mass opt-outs. This issue was especially concerning for participants in countries where an opt-out for the primary use of health data is already in place or will soon be introduced, as they feared that widespread opt-outs could disrupt healthcare delivery and limit the completeness of medical records. Additionally, participants warned that political debates surrounding the EHDS could further amplify misinformation, potentially turning the opt-out into a polarizing issue.

To address this, participants advocated for proactive dissemination of clear, reliable information and education to ensure citizens make informed decisions about the EHDS and the opt-out system, noting that those with poor digital health literacy or low institutional may be more likely to opt-out, potentially skewing data representativeness. One participant described efforts to publish trustworthy resources to counter misinformation about the EHDS:

“So for now, we’re just building the base and just talking about, just making sure all of that information is out there and not from articles that are like, oh my god, the EU is stealing my data” (P27).

Many also emphasized the importance of communicating that health data will largely be anonymized, arguing it could help build public trust. However, others argued that citizens’ concerns about the secondary use of health data extend beyond anonymization. Some participants pointed out that even when data is anonymized, its reuse of health data can still reveal information about specific groups, potentially affecting individuals indirectly. They cautioned that focusing solely on anonymization might not fully address public concerns:

“Uhm, like in more than just anonymizing because this idea, again, are really false idea, that this idea that once data has been anonymized, there’s no more risk to the individual. This is completely false and not the way in which people think about their data” (P1).

### 3.2 Engaging citizens: strategies and challenges

Participants stressed the need to tailor engagement strategies to different groups, particularly distinguishing between patients and the general public. They noted that individuals without significant health concerns—such as younger people—may be less inclined to share their health data, often viewing it through the lens of personal benefit rather than collective wellbeing.

Crucially, participants stressed that engagement efforts must be undertaken with respect to individuals and must be purpose-driven.

“(…) you absolutely have to emphasize is the kind of active engagement and it needs to be, not just tokenistic, it has to be really meaningful engagement with the entire system” (P13).

In this sense, some participants advocated for involving citizens not just as recipients of information but as active contributors to shaping the implementation of the EHDS. They suggested that engaging citizens in discussions about contentious issues—such as overriding the opt-out for public interest purposes—could help align policies with public values and build trust. However, others pointed out that, in their respective countries, there was little tradition of citizen involvement in policy implementation, making bottom-up engagement unlikely. Moreover, others also emphasized the need to respect individuals’ right not to be informed or engaged. They noted that while not every citizen needs to be actively engaged in the secondary use of health data, this passivity should not come at a cost and institutions should ensure that those who choose not to engage are still adequately protected.

“(…) sometimes in the Brussels bubble, people want to, sorry, they want to see the citizen as a sort of a super active and super participant. But people are busy with their lives, you know? They want us to make it simple for them. And also they want us to make it trustworthy enough they don’t have to worry about it” (P8).

Participants also expressed mixed views on citizen participation in the governance structures of the EHDS. Many were unsure how to ensure citizen involvement was meaningful and the extent to which efforts should be made to engage them. Some also voiced concerns about the involvement of patient representatives and organizations, arguing that these groups can sometimes be biased toward their specific diseases and interests:

“(…) but those groups are highly politicized as well. And so it’s going to depend a lot. If you’re talking to a patient organization focusing on rare diseases, you’re going to get completely different answers compared to the patient organization working on cancer” (P1).

Participants emphasized the vital role of patient representatives in promoting the EHDS and engaging the public. These representatives, having first-hand experiences with the benefits of secondary health data, were seen as powerful advocates. Their positive experiences—often tied to improved healthcare outcomes—position them as effective messengers for informing citizens and therefore reducing opt-outs.

Similarly, healthcare professionals were recognized as highly trusted stakeholders, making them essential for engaging citizens. Many participants suggested that securing the support of healthcare providers is critical for EHDS engagement efforts, as their general trustworthiness and direct patient interactions make them more effective in building trust than government or administrative bodies.

“(…) people generally have a good trust in the healthcare provider in the doctor, the nurse and so. But they don’t have such a good trust in the government and in the healthcare administrators” (P18).

One participant highlighted an example from their country where physicians actively campaigned against the introduction of an electronic health record system, leading to a higher-than-expected number of opt-outs. This experience underscored the importance of securing healthcare professionals’ support to prevent a loss of citizens’ trust.

### 3.3 Demonstrating the value of the EHDS to citizens

Participants highlighted the need to demonstrate the tangible benefits and societal value of the secondary use of health data. They stressed that citizens must see a meaningful return on the use of their data, seeing this as essential to fostering trust and reducing resistance.

“I think, especially in (country), where we make this cultural change from opt-in to opt-out, it is super important that we can also prove and show that the European health data space actually does something back for society because that’s the only way to get trust also from our citizens. So I think for (country) at least, it is a very important topic. I think we have to really look into how can we build an ecosystem, an EHDS ecosystem that actually encourages, but also proves the return to society especially when it comes to big companies, as I don’t know Big Pharma, for instance, using their data” (P7).

Another participant highlighted that the decision to opt-out is closely tied to the perception of value creation. They argued that without a clear and compelling explanation of how the EHDS benefits both individuals and society, encouraging participation will be challenging.

“So when we talk about the EHDS (…), without engaging citizens, without social innovation, without describing the value we are able to provide together for everybody, it won’t succeed” (P10).

Participants expressed concerns about a potential imbalance between public and private benefits under the EHDS. While some saw the EHDS as a platform for societal advancement, others worried private companies, such as pharmaceutical firms, would disproportionately benefit from data access. Moreover, many regretted that the regulation lacked specific requirements to guarantee open-source availability of research outcomes and questioned whether public value would be prioritized.

“And then the question is whether in general, you can demonstrate that the public value it creates is more than sort of the privately appropriated value that it creates. And we would hope that if we happen to end up being sort of the HDAB (…), that we are not only servicing super motivated rich private sector companies with health data, but that it’s actually something that helps healthcare research and that helps planning and where we can also be creative in, for instance, accessing privately held data from health care providers to get more patient-reported data into the system and evaluate on that basis” (P20).

Some viewed the secondary use of health data as a means to advance health research and drive innovation, with broad societal benefits such as improving public health. However, they acknowledged the challenge of fostering a sense of solidarity among citizens. They stressed the importance of communicating how collective contributions lead to meaningful long-term benefits, even if these benefits are not immediately visible to individuals.

“How can we make sure that people understand that even though it’s very insignificant, your little piece of information is important together with the rest of us and that will in the future give results for your, I don’t know, children, grandchildren because it takes that long time and that can be frustrating itself” (P6).

Others argued that beyond emphasizing societal-level benefits, such as improved research opportunities, it was necessary to address individual-level value– the “what’s in it for me”– to convince citizens of the EHDS’ value.

“Always the secondary use of the data the question is: why? Why should I consent? Because what is the benefit for me?” (P17).

To make the value of secondary use under the EHDS clear, participants suggested initiatives such as building dedicated websites showcasing data-driven outcomes, organizing national conferences, linking datasets to academic publications, press releases showcasing successful use cases, and engaging with civil society organizations. However, despite these efforts, many acknowledged that societal-level benefits might remain abstract compared to the tangible, individual benefits of primary health data use, such as accessing one’s own health record.

## 4 Discussion

Our study revealed different perspectives among experts on how citizens should be involved in the governance of secondary use. Some emphasized the importance of individual control mechanisms, such as the opt-out, while others argued for structured engagement mechanisms, such as representation and involvement in governance bodies. Additionally, participants underscored the need for clear communication and demonstrating value creation, ensuring that citizens not only understand how their data is used but also perceive tangible benefits from participation.

Citizens can exercise individual control over the secondary use of their health data through the opt-out mechanism in the regulation. While the adoption of this right has generally been welcomed, some MS—including Finland, Denmark (both of which voted against the regulation in the Council) (35) and Estonia—remain dissatisfied. Estonia, for example, in a statement made to the Permanent Representatives of the Committee/Council following the first reading of the legislative act on the 14^th^ January 2025, expressed concerns the opt-out could compromise data quality and completeness, thereby weakening scientific research and innovation. They stated:

“We remain concerned that the obligation to Member States to provide the right to opt out from the secondary use of health data is not in line with the objectives of this Regulation and does not ensure the right balance between individual rights and common public interests. While citizens’ rights and fundamental freedoms need to be protected at any time, there are important safeguards foreseen in EHDS Regulation that can be put in place to ensure that data processing is lawful, secure and in compliance with the GDPR. The introduction of a general opt-out would not contribute to greater data security but entails a risk of eroding the quality and completeness of datasets that are necessary for high quality scientific research and breakthrough innovations” (36).

In addition, Finland and Denmark also highlighted in their statements in the Council that they expect the implementation of the opt-out to be technically complicated (36). This was also reflected as a concern in our study, as there was unclarity over how the opt-out could be implemented across all the health data holders in MS, given that citizens’ health data is distributed across multiple registries and healthcare institutions. It seems further guidance will be needed for MS to adhere to the requirement to provide an “easily understandable and accessible user-friendly mechanism to exercise that right to opt out” (EHDS, Recital 54). It is crucial to prevent the opt-out from becoming an “empty gesture,” as seen with the NHS COVID-19 contact tracing app, which misleadingly continued collecting and sharing data even after individuals opted out (37).

While not explicitly stipulated in the regulation, it seems logical that MS should strive for harmonization in both the formulation and mechanism of the opt-out process. Aligning these aspects as closely as possible would help ensure that citizens across the EU have equal access to this right, preventing disparities that could lead to unequal treatment. Decisions on how to inform citizens about their right to opt-out, how to formulate the opt-out question and how citizens will be able to exercise this right need to be made early on to ensure they meets the requirements under the regulation and fully uphold and respect citizens’ rights. This harmonization could be facilitated through the HDABs Community of Practice (38), which brings together competent authorities and affiliated entities from EU MS and EEA countries.

Beyond affording citizens’ individual control over their health data, the EHDS establishes specific obligations to engage with citizens in the governance of secondary health data use. This includes participation in key bodies such as HDABs, [EHDS, Article 55 (4)] the EHDS Board [EHDS, Article 94 (2)(c)] and the steering groups of HealthData@EU [EHDS, Article 95 (6)]. Additionally, the regulation mandates engagement with citizens for the purpose of supporting digital health literacy and the development of relevant competencies and skills [EHDS, Article 84 (1)]. Citizens could further contribute to other existing health data governance bodies that are likely to closely interact with the EHDS, such as data intermediaries, data cooperatives and data altruism organizations. Likewise, MS which choose—or are required by national law— (EHDS, Recital 37) to incorporate the expertise of ethics committees in their HDABs, may plausibly integrate inputs from citizens and patients, who are often members of these committees. Such kind of patient and public involvement would follow current trends to actively incorporate these voices into public institutions, such as the European Medicines Agency (EMA) (39).

Ensuring meaningful citizen participation, however, requires more than governance structures—it also depends on clear and effective communication. Citizens may not understand terms such as “secondary use,” and a lack of clear information—or worse, the spread of misinformation—could lead citizens to opt-out. Some have suggested using “positive communication” strategies to emphasize the benefits of secondary use (40), while others have stressed the role of mass media and the press in promoting success stories of research outcomes (41), especially given that health data sharing in media narratives is often associated with data breaches or misuse (42–45). As revealed by Understanding Patient Data, “the media typically only discusses the process of using health data when something goes wrong” (46).

However, effective communication requires a careful balance. As reflected in our study, there is a perceived risk that if citizens receive too much information—or if it is framed in a way that overwhelms them— it may generate anxiety, and potentially large-scale opt-outs. This highlights the need to design communication strategies that are informative yet not exaggerated, ensuring that citizens are well-equipped to make informed decisions about their health data without feeling overburdened. MS should build on existing research in the field of science communication to develop plain language communication strategies to inform citizens about the EHDS and the uses of health data (47). Careful consideration should be given to the differing levels of digital access and literacy among various groups of citizens to ensure that information effectively reaches everyone (48).

To address this challenge, some participants in our study suggested that healthcare professionals, such as physicians or nurses, could help communicate information about the use of health data in the EHDS to citizens, especially given that experts perceived them to be highly trusted by citizens. However, this approach requires careful consideration, as it risks damaging patient trust if individuals feel that their doctors’ duty of confidentiality is being compromised due to promoting secondary uses. In fact, the Standing Committee of European Doctors (CPME) called for stronger protections for medical confidentiality, which were finally incorporated into the regulation (EHDS, Recital 24). Additionally, healthcare professionals have raised concerns that the EHDS could increase their administrative burden (49), so suggestions for them to take on this communication role with patients may be unwelcome and fuel resistance to the regulation. This concern is not unfounded—in the UK, a plan to share medical data was rejected by healthcare practitioners, who opposed the expectation that they should communicate this information to patients, citing excessive workload pressures (50).

More broadly, HDABs should develop public websites to provide citizens with clear up-to-date information about data requests, data access permits, available research results and penalties to non-compliant data holders and data users, among other things (51).

While clear communication is essential to meaningfully engage with citizens, developing effective strategies for engagement also requires a deeper understanding of the actual concerns that individuals have about the secondary use of their health data. As our study reveals, experts often assume that citizens’ primary concerns revolve around reidentification, often dismissing these concerns by arguing that the risks for reidentification are low (52), despite the EHDS acknowledging its significance (EHDS, Recital 92) and ongoing debates about the ability to meet anonymization requirements for health data (53), especially genomic data, under the GDPR (54). However, empirical studies suggest that individuals may have reservations about how their health data is used, even when it is anonymized (55). Indeed, some scholars have argued that health data uses can reveal group-level information, which extend beyond individual-level re-identification concerns (56). As Metcalf and Sadowski highlight:

“Data can be harmful even when decoupled from personal identity, and the people and communities which share data about themselves should have a stake in shaping how they’re used even when their data are de-identified” (57).

This concern over group interests was illustrated in a recent controversy involving anonymized UK Biobank data, which was used by a pseudo-scientific group with racist intentions (58). In response, a statement by the European Society of Human Genetics emphasized the importance of ensuring “that access procedures are governed by robust and transparent processes, including about how decisions are made on whether or not the proposed research is in the public interest” (58). This case highlights that citizens’ concerns extend far beyond reidentification risks—misuse remains a serious issue even when citizens cannot be individually identified.

Furthermore, the extent of citizen engagement needed across countries will likely depend on the level of trust that citizens have in public institutions, which will likely be different across MS due to their unique historical and cultural context. Indeed, Peters et al. argue that government credibility is critical in communicating health information and individuals’ willingness to accept these messages (59). In some countries, public skepticism toward institutions or traditionally heightened sensitivities around data protection are likely to result in greater public scrutiny of the EHDS. For instance, since the regulation’s announcement, Dutch tabloid newspapers have described the regulation as being a “gift” to Big Tech (60), arguing that pharmaceutical companies and technology giants would be the primary beneficiaries (61). Furthermore, the Dutch civil society organization “Privacy First,” labels the EHDS as an “excessive power grab by Brussels” (62). In those countries, proactive citizen engagement is essential—not only to foster informed public discourse but also to counter misinformation that could unfairly shape public opinion about the EHDS.

That said, even in countries with traditionally high levels of institutional trust, public reactions to new regulations can be unpredictable. In Finland, for example, media coverage of the EHDS in 2024 led to a surge of GDPR requests to Findata, as citizens sought to object to the processing of their personal data, resulting in delays to their services (63). This suggests that even in trust-rich environments like Finland (64), citizens may still be reluctant to share their health data for secondary use and choose to opt-out.

Given these complexities, it is crucial for MS to ground their citizen engagement strategies on empirical evidence that captures real input from individuals, whether through direct studies or public deliberation models, such as citizen juries (11, 65, 66). This approach is essential, even when findings challenge experts’ preconceived notions about citizens’ preferences and attitudes toward health data use (51). As our study reveals, health data experts’ perspectives may not always align with the concerns of general citizens. Understanding these nuances will be essential to fostering trust, ensuring meaningful participation, and addressing citizens’ actual worries effectively.

Finally, beyond informing citizens and addressing their concerns, effective citizen engagement should also ensure that individuals are aware of the tangible benefits resulting from the EHDS and how these benefits are returned to society. The EHDS may be expected to bring value in many ways, such as through improved scientific research and innovation. However, as noted by Kilgus et al., “this downstream value creation remains insufficiently explored” (67), particularly in terms of how it may ultimately improve healthcare services.

One way in which citizens may see value in data-sharing is through financial returns to the public healthcare system. Our study and the wider literature suggest that commercial entities could be charged higher fees for data access, with the profits reinvested into healthcare systems. This model is already used in three U.S. states, where commercial entities pay more than academic and non-profit institutions for access to health data (68).

However, since the EHDS mandates that the fees be cost-covering (EHDS, Recital 70) and prohibits profit generation, it would not be possible to see a return in monetary profit through fees. Instead, HDABs could explore alternative mechanisms to ensure that citizens see a return in value. One approach could involve agreements with commercial entities to provide priority access to or discounted prices for any medical devices, treatments or therapies that significantly benefited from access to health data. In a draft white paper by the Council of Europe, the Steering Committee for Human Rights in the Fields of Biomedicine and Health emphasized the need for reciprocity for public investment, arguing that since innovative treatments and technologies rely on publicly provided data, pharmaceutical companies and manufacturers should adopt pricing strategies, such as discounts, to ensure equitable access (69). A precedent for this can be seen in the AI firm Sensyne Health’s royalty share agreement with Oxford University hospitals in a project on finding new drug targets for Asthma through genetic sequencing data (70, 71).

Several civil society organizations have put forward similar suggestions, advocating for conditionalities such as making research results public and requirements to accept accessible price caps for medicines resulting from the use of health data when EHDS-facilitated research leads to commercially developed medicines or treatments (72). The European Public Service Union (EPSU) echoed this call for benefit-sharing in its proposed amendments to the EHDS, stating that:

“The profits generated from the collection or use of health data should benefit Member States’ health systems. This requires transparent and robust regulations to ensure that profits from using data are shared fairly and that the general interest takes precedence over private profit” (73).

Such agreements could complement other initiatives such as discounted access to health data for researchers and students, as currently offered by Findata, Finland’s health data permit authority (74). This aligns with Article 62 of the EHDS, which allows MS to establish reduced fees for public entities, including public sector bodies with a legal mandate in the field of public health and university researchers [EHDS, Article 62 (1)].

## 5 Strengths and limitations

This interview study is strengthened by its large and geographically diverse participant pool. However, participants from Western Europe are overrepresented, making up twice the number of those from other regions. This imbalance is likely due to the sampling approach used but may also reflect the differing levels of interest and involvement in the EHDS across different countries and regions. Additionally, the evolving nature of the EHDS regulation at the time of the study presents both a strength and a challenge—it makes the study particularly timely and relevant, yet it also means that perspectives were shaped in a shifting policy landscape that continues to develop.

Another limitation is the absence of citizen representation, despite the study’s focus on citizen engagement. To fully understand public perspectives, this study should be followed up by empirical research that captures citizens’ views on their involvement in the secondary use of health data, building on the substantial body of existing empirical research in this area.

## 6 Conclusion

The EHDS will have significant implications for the use and re-use of EU citizens’ health data. Given this, it seems essential that citizens, at least those interested, have the opportunity to be meaningfully engaged in the secondary use of their health data. At the individual-level, it is critical that citizens are adequately informed about their right to opt-out, with access to clear, balanced information that presents all perspectives—not just the most extreme or skeptical viewpoints— and ensures that the opt-out process is respected and easily accessible. Furthermore, the collective views of citizens should be adequately represented in governance bodies, with efforts to incorporate real, empirical input to ensure that these bodies reflect and address the actual concerns and expectations of the public. Lastly, citizen engagement strategies should focus on clearly communicating the benefits of secondary use under the EHDS and helping citizens understand the value of the regulation. By fostering citizen engagement, MS can ensure that the EHDS not only protects individual rights but also strengthens public trust in the reuse of health data.

## Data Availability

The datasets generated and analysed during the current study are not publicly available due to the conditions of informed consent provided by the participants, which limit data access to the study authors. Requests to access the datasets should be directed to patricia.cerveradelacruz@ugent.be.
