# Supplementary material for: The European Health Data Space: an opportunity to strengthen citizen rights and engage citizens in health data governance
Source: Front Med (Lausanne). 2026 Jan 20;12:1699941. doi: 10.3389/fmed.2025.1699941 (PMC12864452; doi:10.3389/fmed.2025.1699941)
Supplement: Supplementary file 1 [file Data_Sheet_1.docx]

**Interview guide**

**Institutional and professional information**

- Can you please introduce yourself and tell me how you would define your current role within your organization?
- How long have you been in this role?
- Could you explain the organization’s structure and its involvement in the secondary use of health data? / Could you explain how you’ve been involved in the secondary use of health data through your work at X?

**Experiences and Insights on Secondary Health Data Usage**

- Have you had experience with the secondary use of health data?* What did that experience involve?
  - If yes: How do you think this process could be improved?
  - If no: What has been your impression from speaking with researchers about their experiences with secondary use of health data?

*EHDS definition of secondary use of health data: ‘secondary use of electronic health data’ means the processing of electronic health data for purposes listed in Chapter 4 of the EHDS which include: for public interest reasons, to support public sector bodies in the health or care sector, to produce statistics, for education or teaching activities in health or care sectors, for scientific research purposes, for development and innovation purposes, for the training, testing and evaluation of algorithms and for providing personalized healthcare.

- What are the strengths and successes you've observed in the field of secondary health data use, particularly in your country?
  - Potential follow-up questions: What are some of the technical barriers you’ve experienced in the secondary use of health data?

**Specific issues:**

**Privacy and Data Protection**

- What do you think about the data protection aspects when sharing health data?
  - Could you share a few examples of data protection challenges that you see involved when sharing health data?
- Do you think that the current data protection laws, namely the GDPR, are well-suited for secondary use of health data?
  - Do you think the aims of health data sharing can align with the data protection requirements?
  - How do you think researchers can ensure compliance with data protection requirements? Are there any tools or approaches you think are particularly useful for compliance?

**Control, consent and empowerment**

- What do you perceive is a good tool for citizens to control the secondary use of their health data?
- [If consent not mentioned: Consent is often cited as a way to give citizens control for their data.] What consent model do you see to be most appropriate for the secondary use of health data for research purposes?
  - How do you feel about the use of an opt-out for the secondary use of health data?

[If in agreement] What benefits do you think that the use of an opt-out brings?

[If in disagreement] What risks or harms do you think that the use of an opt-out brings?

- Do you think that the type of health data that will be used makes a difference regarding the use of consent or not?
- Do you think that the fact that data will be shared across borders should make a difference to the consent model that is used?
- Do you think that consent is an important way to empower citizens?
  - - [If answer yes] Can you please elaborate on this?
    - [If answer no] What other ways do you think citizens can be empowered in the secondary use of their health data?
- Making sure that individual are informed is an important aspect of research in general. Do you think the current EHDS proposal guarantees a sufficient amount of information provision for individuals?

**Determining public interest (follow-up from legal bases discussion)**

- The EHDS proposal permits the secondary use of health data when this is in the public interest. What do you understand to be in the public interest?/What aspects do you think make a secondary use of health data be in the public interest?
- Understandings of public interest seem to be closely bound to national culture. Do you foresee that we could come a common EU notion of public interest? And how do you think this could be done?
- Is evaluating the public interest of a research project something your organization already does for research projects?
  - - If so, how do you do this?
    - Could you give me a few examples of health data research that you consider to be in the public interest, and conversely, those you believe are not?

**Citizen engagement and trust (follow up from consent)**

- Do you think it is important to engage citizens in the secondary use of health data?
  - Does your organization currently have any citizen engagement initiatives?
  - [If yes] How do you think some of these could be extended to engage citizens within the EHDS?
  - [If no] Why do you think that is?
- The proposed European Health Data Protection Board is expected to invite patient advocacy groups and citizen representatives to sit with them. What is your perspective on the role of these groups in decision-making processes?
  - Do you anticipate involving patient advocacy groups in your organization's decisions, and do you think health data access bodies should be obligated to include these groups in their decision-making?
- How do you expect to communicate secondary uses of health data with citizens?
  - - Do you think this is an important role of your organization?
    - Or do you think that this responsibility lies elsewhere?
- Do you think that the EHDS will enhance individuals rights with regards to the secondary use of health data?
  - - [If yes] What provisions do you think enhance individuals rights?
    - [If no] Why do you think that?
- What elements of the EHDS proposal do you believe can enhance trust among citizens regarding health data sharing, and which elements might negatively impact trust?
- How do you think researchers can build trust with citizens regarding the secondary use of health data?

**Benefit sharing, communication of results and commercial interests**

- Do you think that there is an obligation to return benefits to individuals who shared their health data?
  - - If so, what type of benefits do you think individuals should receive?
- The EHDS requires that data users make the results of their research available to the general public. Do you think results should also be communicated individually?
  - - What about when the research reveals incidental findings? Should this information be communicated to individuals?
    - Do you think any other information should be shared with individuals?
- Do you think that the involvement of commercial or private actors in health data research raises any special concerns?
  - If so, what are they and why do you think they are unique?
- Relating to our earlier discussion about benefit sharing, do you think that private actors should demonstrate a return of investment?
  - If so, what do you think this should look like?

**AI and Health Data (Data representativeness)**

**Question to transition to AI: Do you have any views about AI and how this may impact the secondary use of health data?**

- How do you expect the increasing use of AI in research to impact the secondary use of health data?
- Do you think the use of AI makes a difference with regards to privacy concerns that individuals have?
  - - Do you think that the use of AI should be explicitly communicated to individuals?
    - If so, what information do you think individuals should know?
- AI has been found to exacerbate bias and heighten health inequities. Do you think that the secondary use of health data for the training, testing and development of AI should bear stricter protections?
  - - If so, what additional requirements do you think the secondary use of health data for AI training and development should comply with?
- It is well-reported in the literature that certain population groups are under-represented in the health data used to train algorithms. Do you think that EHDS will improve this situation?
  - How do you think researchers can be encouraged to ensure that their research is representative?
  - What measures do you think that your organization and others can take to improve representativeness and inclusivity in health data research?
- Do you think health data access bodies should be tasked with assessing the risks and benefits associated with a project developing AI with health data?
  - - If not, who do you think should perform this task?

**Future Outlook: EHDS Implementation**

- Considering everything we discussed at the start regarding the existing barriers for the secondary use of health data, how do you think the EHDS will change the situation? Do you think it will improve it? Make it worse? Stay the same?
- Do you think the EHDS will bring further legal clarity?
  - If so, why? (Or, why not?)
  - What things from the regulatory standpoint do you think remain unclear?
  - The EHDS highlights the importance of upholding the FAIR data principles (meaning that data be findable, accessible, interoperable and reusable). What has been your experience with these principles? Have you ever found the meaning to be misunderstood to deal with issues of fairness in data use, rather than with the technical and organizational issues?
- How do you see your current role changing with respect to the upcoming EHDS?
  - What new responsibilities do you expect the EHDS to bring for your organization?
- Are there any issues you expect to remain even after the implementation of the EHDS?
  - Are there any things beyond the regulation that you think are needed to facilitate the secondary use of health data?

**Concluding question:**

Is there anything else you would like to add that we have not covered in this interview?

Finally, do you know of any other expert that would be interested to discuss the topic during an interview?

**Interview Guide Checklist**

**1. Institutional and Professional Information**

- Introduction of the interviewee and definition of their current role.
- Duration of the interviewee's current role.
- Explanation of the organization's structure and involvement in the secondary use of health data.

**2. Experiences and Insights on Secondary Health Data Usage**

- Experience with secondary use of health data.
  - If yes: Suggestions for improvement.
  - If no: Impression from speaking with researchers.
- Strengths and successes observed in secondary health data use.
- Technical barriers experienced in secondary use of health data.

**3. Privacy and Data Protection**

- Thoughts on data protection aspects when sharing health data.
  - Examples of data protection challenges.
- Suitability of current data protection laws (e.g., GDPR) for secondary use.
  - Alignment of health data sharing aims with data protection requirements.
  - Approaches for ensuring compliance with data protection requirements.

**4. Control, Consent, and Empowerment**

- Perception of tools for citizens to control secondary use of health data.
- Opinion on consent models for research purposes.
- Views on using opt-out for secondary use of health data.
- Consideration of data type and cross-border sharing in consent models.
- Importance of consent in empowering citizens.
  - Elaboration on why consent is empowering or alternative empowerment methods.
- Assessment of EHDS proposal in providing sufficient information to individuals.

**5. Determining Public Interest**

- Evaluation of public interest in health data access bodies.
  - Aspects defining public interest in secondary use of health data.
- Existing practices of evaluating public interest in research projects.
  - Examples of health data research considered/not considered in the public interest.

**6. Citizen Engagement and Trust**

- Importance of engaging citizens in secondary use of health data.
- Existence of citizen engagement initiatives in the organization.
  - Suggestions for extending initiatives within the EHDS.
- Perspective on the role of patient advocacy groups in decision-making.
- Expectations regarding involvement of patient advocacy groups in decision-making.
- Strategies for communicating secondary uses of health data with citizens.
  - Views on the EHDS enhancing individual rights and trust.

**7. Benefit Sharing, Communication of Results, and Commercial Interests**

- Consideration of obligations to return benefits to individuals.
  - Types of benefits individuals should receive.
- Thoughts on communicating research results individually.
  - Handling incidental findings and other shared information.
- Concerns and unique aspects of involving commercial or private actors in health data research.
- Opinions on private actors demonstrating a return on investment.

**8. AI and Health Data (Data Representativeness)**

- Views on AI's impact on the secondary use of health data.
  - Expectations regarding AI's influence on privacy concerns.
  - Opinions on explicit communication of AI use to individuals.
- Consideration of stricter protections for health data used in AI training.
  - Additional requirements for AI training and development with health data.
- Expectations on EHDS improving data representativeness.
  - Strategies for improving representativeness and inclusivity.
- Opinions on health data access bodies assessing risks and benefits in AI projects.

**9. Future Outlook: EHDS Implementation**

- Assessment of EHDS impact on existing barriers.
  - Expectation of improvement, worsening, or remaining the same.
- Views on EHDS bringing legal clarity.
  - Identification of unclear regulatory aspects.
- Anticipation of role changes with EHDS implementation.
  - Expected new responsibilities.
- Identification of potential issues post-EHDS implementation.
  - Consideration of additional measures beyond regulation for facilitating health data use.
